# Protein diffusion in plant cell plasma membranes: the cell-wall corral

**DOI:** 10.3389/fpls.2013.00515

**Published:** 2013-12-17

**Authors:** Alexandre Martinière, John Runions

**Affiliations:** ^1^Biochimie et Physiologie Moléculaire des Plantes, Centre National de la Recherche Scientifique, Institut National de la Recherche Agronomique, Université Montpellier 2Montpellier, France; ^2^Department of Biological and Medical Sciences, Oxford Brookes UniversityOxford, UK

**Keywords:** lateral mobility, plasma membrane microdomains, diffusion, corral, cell wall, cytoskeleton

## Abstract

Studying protein diffusion informs us about how proteins interact with their environment. Work on protein diffusion over the last several decades has illustrated the complex nature of biological lipid bilayers. The plasma membrane contains an array of membrane-spanning proteins or proteins with peripheral membrane associations. Maintenance of plasma membrane microstructure can be via physical features that provide intrinsic ordering such as lipid microdomains, or from membrane-associated structures such as the cytoskeleton. Recent evidence indicates, that in the case of plant cells, the cell wall seems to be a major player in maintaining plasma membrane microstructure. This interconnection / interaction between cell-wall and plasma membrane proteins most likely plays an important role in signal transduction, cell growth, and cell physiological responses to the environment.

## Introduction

Due to thermal agitation, molecules diffuse within liquid environments. In the specific case of proteins anchored within membranes, their diffusion is restricted to the two dimensions of the membrane plane. This type of motion is called lateral mobility. The propensity of a protein to move within the plane of a membrane has important biological consequences in terms of membrane micro-organization, protein-protein interactions and signal transduction mechanisms. An example of this is receptor clustering within the plasma membrane (PM) of post-synaptic neurons, where lateral mobility is linked to nerve plasticity (for review see: Choquet and Triller, 2003). In plant biology, thanks to recent microscopical advances, links between spatial segregation of proteins and signal transduction have been made. For instance, abscisic acid signaling via inhibition of ABI1 (abscissic-acid instensitive 1) modulates, within micro-domains, the recruitment of a complex between the anion transporter SLAH3 (slow anion channel 1 homolog 3) and its regulatory calcium dependent protein kinase (CPK21) (Demir et al., 2013). Similarly, a recent study has shown that, upon ammonium treatment, ammonium transporter 1.3 (AMT1.3) forms clusters within the PM prior to activation of an endocytotic mechanism. This suggests that AMT1.3 dynamics play a functional role in the cell's response to NH^4+^ (Wang et al., 2013). These results illustrate the central role of protein lateral mobility in plant cell responses to their environment.

## FRAP and other approaches

One of the most popular approaches for study of protein lateral mobility is Fluorescence Recovery After Photobleaching (FRAP). FRAP is simple to do and yields results quickly when working with living cells. Membrane proteins tagged with fluorescent proteins are especially amenable to this technique. Fluorescence is bleached by a laser beam within a Region of Interest (ROI). Reappearance of fluorescence in the bleached ROI is then monitored over time. Mean fluorescence intensity within the ROI increases if fluorescent molecules are free to diffuse into it from the non-bleached surrounding region during the post-bleaching phase. This increase in mean fluorescence intensity can be fit with curve equations to yield information of the type of diffusion under study (Axelrod, 1983; Feder et al., 1996; Sprague et al., 2004). For example, one could ask, is the protein under study free to diffuse or is it constrained in its lateral mobility by interaction with another cellular structure? Two important quantitative values arise from the equation of a recovery curve fit to a set of FRAP data, and these describe the amount and rate of protein lateral mobility under study. The first of these, the “mobile fraction,” describes the fraction of a given protein that is unconstrained and therefore free to diffuse within the membrane. Secondly, the “half time” (*t*_1/2_) describes the rate at which diffusing molecules do so. Depending of the shape of the ROI, *t*_1/2_ can be used to extrapolate a relative diffusion coefficient (Yang et al., 2010; Mai et al., 2013). Calculated “half times,” are useful for comparing mobilities of different proteins.

Interestingly, the fluorescence recovery curve of PM proteins describes the sum of at least two additive mechanisms: the lateral mobility of the protein within the PM, and the exchange of proteins between cytoplasmic vesicles and the PM by endocytosis and exocytosis. It is possible to distinguish between protein exchange on and off the membrane, and lateral diffusion by analysing fluorescence recovery images with the help of 1D Gaussian fits. To do this, fluorescence intensity is measured along a line fit through the center of the bleached region and plotted against distance from the center of the bleached spot. In the case of pure lateral diffusion in which fluorescence recovery is only possible from the margins of the bleached region, the Gaussian profile becomes more shallow and widens over time so that the area under the curve is conserved. Conversely, for the case of fluorescence recovery that is caused by protein addition to the membrane by exocytosis, the Gaussian curve maintains its width over time while its area is reduced. This is because exocytosis happens uniformly over the entire bleached region (Oancea et al., 1998; Hammond et al., 2009; Luu et al., 2012).

Recent advances in microscope technology and new fluorescent protein development now allow imaging of single fluorescent molecules in living membranes and consequently their dynamics are directly visible. These approaches have been successfully used on plant samples allowing recording of diffusion coefficients of PIP proteins (Li et al., 2011), and of the PM marker paGFP-LTI6b (Martinière et al., 2012). Super resolution microscopes which are capable of imaging at sub-diffration-limited dimensions have recently been used to image PIN auxin carrier recycling at the PM (Kleine-Vehn et al., 2011) and will change our vision of plant protein dynamics as has already happened in the neurosciences (Maglione and Sigrist, 2013).

## Protein lateral mobility in the plant plasma membrane

Our knowledge about the lateral mobility of plant PM proteins is increasing very quickly. One of the first reports was on dynamics of the potassium channel KAT1 (Sutter et al., 2006). In that study, a fusion between KAT1 and photoactivable-GFP was used to record the lateral mobility of this protein. Measurement of protein dynamics using photoactivatable fluorescent proteins is like FRAP flipped on its head. The ROI starts out at 100% brightness after photoactivation and diffusion results in a decrease in its mean fluorescence intensity over time. In the case of KAT1, ROI fluorescence did not appreciably decrease, even after several minutes, suggestive of a very small mobile fraction; in other words, the potassium channel seems to be immobile within the PM. This very counterintuitive result was later confirmed for other PM proteins. Men et al. (2008) demonstrated that after 20 min PIN2-GFP only recovers to 40% of its pre-bleach intensity within an ROI. Similarly, FRAP experiments on BOR1, KNOLLE, NIP5.1, and CASP1 suggest very low lateral mobility of these proteins (Table 1) (Boutté et al., 2010; Takano et al., 2010; Roppolo et al., 2011).

**Table 1:** **Mobile fraction of proteins evaluated by FRAP**.

**Constructs**	**Expression system**	**Mobile fraction %**	**Time of observation**	**References**
GFP-Lti6b	Stable line p35S	76	120 s	Kleine-Vehn et al., 2011
	Transient expression, p35S	84	60 s	Martinière et al., 2012
	Stable line p35S	96	60 s	Martinière et al., 2012
PIN2-GFP	Stable lines pPIN2	13	60 s	Feraru et al., 2011
		>20	200 s	Men et al., 2008
		14	120 s	Kleine-Vehn et al., 2011
PIN1-GFP	Stable lines pPIN1	17	120 s	Kleine-Vehn et al., 2011
PIP2;1-GFP	Stable line p35S	10	60 s	Luu et al., 2012
	Stable lines p35S	10	60 s	Martinière et al., 2012
PIP2;1-CFP	Transient expression, p35S	43	60 s	Martinière et al., 2012
PIP1;2-GFP	Stable line p35S	10	60 s	Luu et al., 2012
GFP-NPSN11	Transient expression, p35S	10	60 s	Martinière et al., 2012
GFP-AGP4	Transient expression, p35S	20	60 s	Martinière et al., 2012
GFP-StREM1.3	Transient expression, p35S	23	60 s	Martinière et al., 2012
AtFLS2-GFP	Stable lines, pFLS2	19	60 s	Martinière et al., 2012
GPa1-GFP	Transient expression, p35S	79	60 s	Martinière et al., 2012
AtFH1-GFP	Transient expression, p35S	18	60 s	Martinière et al., 2012
YFP-AtSYP121	Transient expression, p35S	23	60 s	Martinière et al., 2012
At1g14870-GFP	Transient expression, p35S	36	60 s	Martinière et al., 2012
At3g17840-GFP	Transient expression, p35S	58	60 s	Martinière et al., 2012
BOR1	Stable lines	<40	20 min	Takano et al., 2010
mCitrin-NIP5;1	Stable lines	<50	20 min	Roppolo et al., 2011
GFP-KNOLLE	Stable lines, pKNOLLE	>60	10 min	Boutté et al., 2010
CASP1-GFP	Stable lines, pCASP	<15	20 min	Roppolo et al., 2011

As explained earlier, lateral diffusion of a protein is a direct consequence of temperature and its diffusion constant is dependent mainly on a protein's hydrodynamic radius and the viscosity of the membrane (Saffman and Delbrück, 1975). Numerous examples show high mobile fractions and diffusion coefficients from 0.1–1 μm^2^/s in animal cells (for review Owen et al., 2009). Plant cells are proving to be somewhat variable. For instance, GFP-AQP1 expressed in LLC-PK1 cells recovers to 100% pre-bleach brightness after 2 min (Umenishi et al., 2000) while GFP-PIP2.1, a plant homologue of AQP1, recovers to only 10% of its pre-bleach fluorescence after the same time (Luu et al., 2012). Many proteins in the plant PM seem to be constrained in their diffusion by mechanisms not found in animal cells. Sorieul et al. (2011) have compared the recovery curve of two AtPIP2;1-GFP constructs, one in the PM and the other carrying point mutations which cause it to be retained within the endoplasmic reticulum. Very low lateral mobility was only observed when the aquaporin is in the PM. This does not result from higher viscosity of plant PMs relative to other endomembranes like the ER because some proteins like LTI6b-GFP have high mobile fractions in the same conditions (Table 1) (Kleine-Vehn et al., 2011; Luu et al., 2012; Martinière et al., 2012).

## A cell wall corral

A central question in our research, then, is how and why are some PM proteins fixed in place while physical laws would dictate that they move. Two explanations arise. First, PM proteins could, via a specific interaction, be bound to a surrounding structure in the vicinity of the PM. For instance, PM-anchored protein A could bind to B which is a non-diffusible object or itself attached to a non-diffusible object, consequently limiting protein A diffusion. Alternatively, or in addition, PM proteins might have constrained mobility due to steric hindrance. In other words, protein lateral mobility is restricted due to crowding with other PM proteins that themselves are involved in interactions with other cellular constituents. Both explanations have been demonstrated for plant cells. In the case of Arabidopsis Formin1 (AtFH1), low lateral mobility is due to a specific interaction between the extracellular domain of the protein and a cell wall component, most likely mediated through an extensin binding motif of AtFH1 (Martinière et al., 2011). Low lateral mobility of other PM proteins might be the result of direct interactions with the cell wall or the cytoskeleton. This is perhaps the case for the WAK protein family that interact with oligogalacturonides of the cell wall (Steinwand and Kieber, 2010). Similarly, it is tempting to think that families of RLKs (receptor-like kinases), which are involved in cell wall-sensing and have large extracellular extensions, are directly linked to cell-wall constituents (for review see: Hématy and Höfte, 2008).

As stated earlier, compared to animal cells, many plant PM proteins have far smaller lateral mobilities. Nevertheless, animal PM proteins rarely exhibit free diffusion behavior. It is, in fact, common for them to have substantially altered motion. In the past 40 years, the fluid mosaic model proposed by Singer and Nicolson (1972), in which membrane components are uniformly distributed, has slowly evolved to a model in which membranes are composed of a multitude of microdomains (Engelman, 2005; Nicolson, 2013). Microdomains are maintained over time either by a heterogeneity in PM composition, e.g., as lipid microdomains, or by corrals formed of cytoskeletal elements in close proximity to the PM which limit movement of PM proteins which project into the cytoplasm (Tomishige et al., 1998). In both cases, these structures constrain lateral mobility of PM proteins. Very well documented examples show a partitioning of proteins between microdomains in, e.g., raft-, and non-raft fractions, and that microdomain organization of membrane proteins can be linked to signal transduction mechanisms (review in Simons and Ikonen, 1997; Simons and Gerl, 2010). PM proteins can exhibit various types of lateral mobility. In Li et al. (2011), fluorescent variants of GFP-PIP2;1 have been described with three motion modes: Brownian, directed, and restricted (reviewed in Owen et al., 2009). Brownian motion is typical for objects with random trajectories. Directed motion means that particles are moved via energetic processes such as in the case of myosin-mediated movement along the actin cytoskeleton. Finally, restricted motion happens when particles are confined in a small area of the membrane such as within a lipid microdomain. Interestingly, lateral mobility of GFP-PIP2;1 seems to involve all three types of motion. It is known that some minutes after addition of a salt stress, PIP proteins undergo rapid and quantitative endocytosis (Luu et al., 2012). Examination of the early events in this process found that PIP2;1 has a tendency to be restricted in its mobility. When salt treatment was combined with drug treatments to alter trafficking, PIP2;1 changed from being immobile to restricted motion and then was endocytosed. This result suggests that, as in animal cells, plant PMs have microdomain organization, e.g., lipid microdomains and/or corralling cytoskeletons which modulate protein mobility (for review see: Malinsky et al., 2013).

Lipid microdomains in plant cells have been biochemically characterized for several species including *Arabidopsis thaliana* (for review Simon-Plas et al., 2011). These microdomains can partition proteins within the plane of the membrane and show a general enrichment for proteins involved in signaling, cell trafficking and cell-wall metabolism (Borner et al., 2005; Keinath et al., 2010). Interestingly, in plant-pathogen interaction, the leucine-repeat-rich receptor kinase FLS2 is recruited into detergent insoluble fraction (DIM) upon elicitation with flg22 (Keinath et al., 2010). This suggests a model in which lipid partitioning plays a role in lateral mobility of plant PM proteins.

A cell's extracellular matrix (ECM) is also an important feature in regulating protein lateral mobility. Research in yeast has shown that the periplasm and the cell wall both modify lateral mobility of lipid probes (Greenberg and Axelrod, 1993). More recently, an extensive work on yeast PM microdomains has shown that membrane micro-organization is perturbed by loss of the cell wall (Spira et al., 2012). Similarly, in nerve cells, the ECM hinders lateral mobility of glutamate receptors (Frischknecht et al., 2009). In plant cells, outward turgor pressure forces the PM to be very tightly appressed to the cell wall. A recent study has show that this intimate connection affects protein lateral mobility (Martinière et al., 2012). Sets of artificial proteins were used to describe the influence of the cell wall mechanism on lateral mobility. Proteins with amino acids projecting into the outer phase of the membrane or into the extracellular space had a low lateral mobility which increased if the cell wall was perturbed or removed (Martinière et al., 2012). Consequently, these results suggest that the plant cell wall, and by extension the continuum between the PM and the cell wall influences protein lateral mobility. This regulation of protein lateral mobility could play a role in myriad cellular processes. For instance, in root tip cells, the polar localization of PIN2 disappeared under plasmolysis treatment or when a weak cell wall digestion was performed (Feraru et al., 2011). The likely explanation for this observation is that the cell wall restricts lateral mobility of PIN2 and consequently helps maintain its polarized localization in root epidermal cells.

## Protein immobility in the PM and its consequences

The relative immobility of most plant PM proteins needs to be looked at in context. Mobile fractions generated from FRAP data are always done on small time scales from seconds to tens of minutes. This time scale undoubtedly has significance in terms of signal transduction, but is maybe less meaningful for longer process during development. However, the polarized localization of auxin transporters such as PIN1 or PIN2 is, at least in part, based on low lateral mobility within the PM. Indeed, only the additive effect of polarized exocytosis and endocytosis coupled with a low lateral mobility allows model to predict with accuracy the localization of PIN proteins (Kleine-Vehn et al., 2011). As a consequence, low lateral mobility of proteins acts in polarized localization of auxin and, therefore, participates in root development.

Recent findings about the Casparian strip are also meaningful in terms of lateral mobility of proteins within the PM. The Casparian strip is a cell wall barrier made in part of suberin and lignin surrounding the endodermis tissue in plant roots (review in Geldner, 2013). This barrier limits diffusion of water and ions between outer layers of the root and its vascular tissue. Interestingly, this barrier also stops totally the diffusion of lipids within the PM of endodermis cells (Alassimone et al., 2010). The molecular processes involved in Casparian strip formation are beginning to be understood. CASP proteins which are localized at first everywhere within the PM are later found only where the Casparian strip will be formed (Roppolo et al., 2011). Strikingly, in terms of lateral mobility, CASP proteins are relatively more mobile at early stages than in later stages of development. This suggests that in the region where the Casparian strip will be formed, CASP proteins are anchored by an unknown mechanism that might include interaction with the cytoskeleton or the cell wall (Roppolo and Geldner, 2012). This example serves to illustrate again the importance of protein lateral mobility in regulation of development processes and clearly show that plant can modify their cell wall composition to influence on diffusion of proteins within their PM.

A mechanism for regulation of cellular process such as signal reception is “control by change in location” (Malinsky et al., 2013). In others words, membrane proteins move laterally to effect activation of a signaling mechanism. This phenomenon has recently been described in the cases of the ammonium transporter AMT and the SAL3/CPIK23 complexes (explained in detail earlier). It is tempting to extrapolate these findings to other signaling cascades, especially the case of flagellin signaling, it is know that FLS2, the receptor for flg22, has its lateral mobility restricted upon interaction with its ligand (Ali et al., 2007). This again suggests an interconnection between dynamic partitioning in the PM and signal transduction mechanisms, even if formal proof is still missing.

## Conclusion and future prospects

FRAP approaches have revolutionized the study of membrane protein dynamics in living cells. These techniques make possible the study of membrane structure and promise to be useful in elucidation of signaling mechanisms.

Many PM proteins have very low lateral mobility which suggests a high degree of membrane organization and a natural tendency of proteins to groups themselves in clusters, e.g., KAT1, StREM3.1, PIN2, AMT1.3, and AtFlot1 (Sutter et al., 2006; Raffaele et al., 2009; Kleine-Vehn et al., 2011; Li et al., 2012; Wang et al., 2013). Bioimaging not only lets us study protein dynamics and association, but now gives us accessible tools for studying protein-protein interactions. Receptor-mediated signaling mechanisms should be observable by combining FRAP techniques with fluorescence resonance energy transfer (FRET) and fluorescence lifetime imaging (FLIM). In FRET, interacting proteins become visible by alterations in their fluorescence emission and this is observable as diffusion is monitored via FRAP.

Super-resolution microscopy techniques such as stimulated emission depletion (SIM), structured illumination (STED) and photoactivated localization microscopy (PALM) are capable of resolving objects 50-70nm in size, far below the current limit for light microscopy of 200-300nm. Coupled with total internal reflection fluorescence microscopy (TIRF), its allow studying the dynamics of individual molecules. This single-molecule tracking technique has been employed to study movement of the protein in plant PM (Li et al., 2011; Martinière et al., 2012). Ongoing work in our labs will seek to combine FRAP for measurement of protein dynamics with higher resolution techniques that will allow finer-scale dissection of the mechanisms involved in membrane transport and pathogen response.

Finally, one of the main findings of our recent work was that the cell wall interacts with and stabilizes PM proteins. We are now engaged in trying to elucidate the mechanism of cell wall—PM protein interaction. One of the approaches to this problem will be to study dynamics of PM proteins in cell-wall mutant backgrounds that are altered in, or lacking different cell-wall components such as pectin and cellulose. The cell-wall may turn out to have functions in cell signaling that we do not yet appreciate.

### Conflict of interest statement

The authors declare that the research was conducted in the absence of any commercial or financial relationships that could be construed as a potential conflict of interest.
